# Inferring pain experience in infants using quantitative whole-brain functional MRI signatures: a cross-sectional, observational study

**DOI:** 10.1016/S2589-7500(20)30168-0

**Published:** 2020-08-24

**Authors:** Eugene P Duff, Fiona Moultrie, Marianne van der Vaart, Sezgi Goksan, Alexandra Abos, Sean P Fitzgibbon, Luke Baxter, Tor D Wager, Rebeccah Slater

**Affiliations:** a Department of Paediatrics, University of Oxford, Oxford, UK; b Wellcome Centre for Integrative Neuroimaging, Oxford Centre for Functional MRI of the Brain, Nuffield Department of Clinical Neurosciences, University of Oxford, Oxford, UK; c Laboratory of Neuroimaging and Cognition, Medical Psychology Unit, Department of Medicine, University of Barcelona, Barcelona, Spain; d Department of Psychological and Brain Sciences, Dartmouth College, Hanover, NH, USA; e Department of Psychology and Neurosciences, University of Colorado, Boulder, CO, USA

## Abstract

**Background:**

In the absence of verbal communication, it is challenging to infer an individual's sensory and emotional experience. In communicative adults, functional MRI (fMRI) has been used to develop multivariate brain activity signatures, which reliably capture elements of human pain experience. We aimed to translate whole-brain fMRI signatures that encode pain perception in adults to the newborn infant brain, to advance understanding of functional brain development and pain perception in early life.

**Methods:**

In this cross-sectional, observational study, we recruited adults at the University of Oxford (Oxford, UK) and infants on the postnatal wards of John Radcliffe Hospital (Oxford, UK). Healthy full-term infants were eligible for inclusion if they were clinically stable, self-ventilating in air, and had no neurological abnormalities. Infants were consecutively recruited in two cohorts (A and B) due to the installation of a new fMRI scanner using the same recruitment criteria. Adults (aged ≥18 years) were eligible if they were postgraduate students or staff at the University of Oxford. Participants were stimulated with low intensity nociceptive stimuli (64, 128, 256, and 512 mN in adults; 64 and 128 mN in infants) during acquisition of fMRI data. fMRI pain signatures (neurologic pain signature [NPS] and stimulus intensity independent pain signature-1 [SIIPS1]), and four control signatures (the vicarious pain signature, the picture-induced negative emotion signature [PINES], the social rejection signature, and a global signal signature) were applied directly to the adult data and translated to the infant brain. We assessed the concordance of the signatures with the brain responses of adults and infants using cosine similarity scores, and we assessed stimulus intensity encoding of the signature responses using a Spearman rank correlation test. We also assessed brain activity in pro-pain and anti-pain components of the signatures.

**Findings:**

Between May 22, 2013, and Jan 29, 2018, we recruited ten healthy participants to the adult cohort (five women and five men; mean age 28·3 years [range 23–36]), 15 infants to infant cohort A (six girls and nine boys; mean postnatal age 4 days [range 1–11]), and 22 infants to infant cohort B (11 girls and 11 boys; mean postnatal age 3 days [range 1–10]). The NPS was activated in both the adults and infants, and reliably encoded stimulus intensity. The NPS was activated in the adult cohort (p<0·0001) and both infant cohorts (p=0·048 for infant cohort A; p=0·001 for infant cohort B). The SIIPS1 was only expressed in adults. Pro-pain brain regions showed similar activation patterns in adults and infants, whereas responses in anti-pain brain regions were divergent.

**Interpretation:**

Basic intensity encoding of nociceptive information is similar in adults and infants. However, translation of adult brain signatures to infants indicated substantial differences in infant cerebral processing of nociceptive information, which might reflect their absence of expectation, motivation, and contextualisation associated with pain. This study expands the use of brain activity pain signatures to non-verbal patients and provides a potential research approach to assess the impact of analgesic interventions on brain function in infants.

**Funding:**

Wellcome Trust, Supporting the Sick Newborn and their Parents Medical Research Fund.

## Introduction

In the absence of verbal communication, the perceptions of pain in infants are not known. Behavioural cues such as grimacing and vocalisation are relied on to make inferences regarding an infant's perception of pain,^1^ which are broadly based on the experiences of older children and adults capable of verbalisation. However, behaviours have poor sensitivity and specificity and interpretation is subjective. Behaviours attributed to pain can be elicited by non-noxious stimuli in premature infants^2^ and some infants have high behavioural pain scores in response to apparently innocuous procedures such as a nappy changing.^3^ Although pain scores such as the Premature Infant Pain Profile-Revised have some construct validity,^1^ the extent to which these scores correspond with pain perception is unknown. Considering that pain perception is encoded by the brain, neuroimaging provides a proxy measure of neural activity that can be compared between infants and adults to make valuable inferences about pain experience in this non-verbal population.

**Research in context**

**Evidence before this study**

We searched PubMed for articles published from inception to April 1, 2020, which included the search terms ‘fMRI’, ‘infant or neonate’, and ‘pain or nociception’ in the title or abstract. As a result of the recent emergence of this field in the past 5 years, and the experimental and analytical challenges involved in studying cerebral processing of pain in the MRI environment in healthy newborn infants, our search only yielded five studies of functional MRI (fMRI) assessing infant brain responses to nociceptive stimuli. In a foundational pilot study, an experimental noxious stimulus was applied to a single infant, which evoked widespread brain activity that included several brain regions involved in pain processing in adults. In a subsequent observational cohort study, regional analyses were used to compare active brain regions in infants (n=10) and adults (n=10), which found that the evoked patterns of brain activity were broadly similar in infants and adults. Further follow-up analysis in the infant cohort showed that the functional connectivity of brain regions involved in descending pain modulation influences the magnitude of pain-related brain activity. Two further studies focused on methodological advances, providing evidence-based recommendations for fMRI acquisition parameters and image processing to maximise the quality of infant data, and these methods have been implemented in this study.

**Added value of this study**

This study translates validated adult pain fMRI brain signatures to a non-verbal patient population in which the assessment and management of pain presents a considerable clinical challenge. Application of fMRI brain signatures to newborn infants expands on previous fMRI studies, which have provided only qualitative evidence that noxious stimulation commonly activates brain regions in the adult and infant brain. In this study, we have shown that the basic encoding of the sensory discriminative aspects of pain, as represented by the neurologic pain signature (NPS), occurs in both adults and infants, whereas higher-level cognitive modulation of pain, represented by the stimulus intensity independent pain signature-1, is only present in adults and not observed in infants. We hypothesise that the differences in how immature infants process pain, relative to mature adults, are likely to reflect differences in their expectation, motivation, and the contextualisation of external events rather than differences in their core nociceptive cerebral processing of pain. The findings of our study enable the use of quantitative fMRI observations to make stronger inferences regarding pain experience in non-verbal infants.

**Implications of all the available evidence**

Behavioural pain scores used in neonatal clinical care have low sensitivity and specificity. Previous clinical trials in newborn babies in which such scores have been used as outcome measures found common analgesic interventions were not effective in reducing behavioural scores, resulting in few evidence-based drugs for treating pain. The potential value of using brain-based neuroimaging markers of pain as a method of providing objective evidence of analgesic efficacy in early proof of concept studies is recognised in adults, even in the absence of behavioural pain modulation. Similarly, in infants, electroencephalogram-based measures of noxious-evoked brain activity have been used as outcome measures in clinical trials of analgesics to overcome some of the inherent limitations of using behavioural observations to quantify analgesic efficacy. Considering the successful translation of the NPS and its sensitivity to analgesic modulation in adults, this novel methodology represents an objective brain-based fMRI approach that could be used to advance the identification and assessment of analgesic interventions for infants.

Pain has no specific nexus in the human brain. A disperse mode of neural activity across sensory, limbic, and other areas, with complex temporal and stochastic characteristics is hypothesised to underlie an individual's subjective perception of pain.^4^ Adult pain involves complex cognitive and emotional processes, which are influenced by anticipation, attention, memory, salience, attribution of causality, fear, and anxiety.^5^ However, many of these modulatory factors might not influence pain perception in early life. As the complexity of structural and functional brain circuitry develops, a wider range of emotional, cognitive, and motivational elements constituting the conscious experience of pain is likely to evolve. Functional MRI (fMRI) has been extensively used to characterise the central nociceptive system in adults, and in infants, noxious-evoked neural activity can be detected.^6^, ^7^ Regional analysis of adult and infant brain responses has shown broadly similar patterns of noxious-evoked activity,^6^ with more bilateral activation observed in infants than adults, potentially as a result of exuberant corticocortical and interhemispheric connectivity of the immature brain. Additional brain regions were also activated in infants, such as the hippocampus and caudate, and no activity was reported in other regions such as the amygdala and orbitofrontal cortex. However, inferring the perceptual consequences of functional activity or inactivity within anatomical regions is challenging.

Regionalised assessments of brain activity can be combined to achieve robust inferences. Multivariate spatial patterns of fMRI activity have been identified that can predict specific aspects of a reported experience.^8^ A series of adult fMRI studies have characterised signatures associated with pain and negative affect.^9^, ^10^, ^11^, ^12^, ^13^, ^14^ The primary pain template is the neurologic pain signature (NPS), which predicts reported pain intensity.^12^ The NPS was trained on responses to heat pain of varying intensity and accurately predicted pain in more than 40 independent cohorts,^8^ including pain elicited by electrical and mechanical^11^ stimuli. The NPS is sensitive to nociceptive pain and does not correspond to activity evoked by non-noxious stimuli,^12^ vicarious pain,^11^ threat,^11^ negative affect,^9^ or social pain (ie, pain as a result of interpersonal rejection or loss).^12^ A second signature, the stimulus intensity independent pain signature-1 (SIIPS1), was developed to explain additional variation in reported pain, associated with modulatory factors such as expectation, motivation, context, and perceived control.^13^ The SIIPS1 signature tracks variability in subjective pain report that is not accounted for by stimulus intensity and the NPS.

Verbal report is the gold standard method of measuring pain in adults, but since infants cannot use language to describe their pain, the identification of objective surrogate measures to quantify pain experience in infants would be valuable. In this study, we aimed to project functional neuroimaging signatures of pain onto evoked brain activity to identify and quantify neural patterns of activity associated with aspects of pain experience in adults and infants. We also aimed to investigate the concordance of the signatures with noxious-evoked brain activity in adults and infants, compare signature expression across stimulus intensities, and assess the responses within pro-pain and anti-pain components of the signatures.

## Methods

### Study design and participants

In this cross-sectional, observational study, we recruited adults at University of Oxford (Oxford, UK) and infants on the postnatal wards of John Radcliffe Hospital (Oxford, UK). Healthy full-term infants were eligible for inclusion if they were clinically stable, self-ventilating in air, and had no neurological abnormalities. Parents were given a description of the study and invited to test the experimental stimuli. Infants were recruited in two cohorts (A and B) using the same criteria. Cohort A was recruited before the installation of a new fMRI scanner and cohort B was recruited after May 25, 2015, following the scanner upgrade. Adults (aged ≥18 years) were eligible if they were postgraduate students or staff at the University of Oxford. Informed written consent was provided by adult participants and by the infants’ parents. MRI data were acquired at the Oxford Centre for Functional MRI of the Brain (FMRIB; University of Oxford, Oxford, UK). Ethical approval was obtained from the National Research Ethics Service for the infant study, and from the University of Oxford Central University Research Ethics Committee for the adult study. The study was done in accordance with the Declaration of Helsinki and Good Clinical Practice guidelines. The Ethics Committees have deemed that potential benefits of the research to the wider population outweigh the minimal potential risk to the infants.^15^

### Procedures

**Figure 1:**
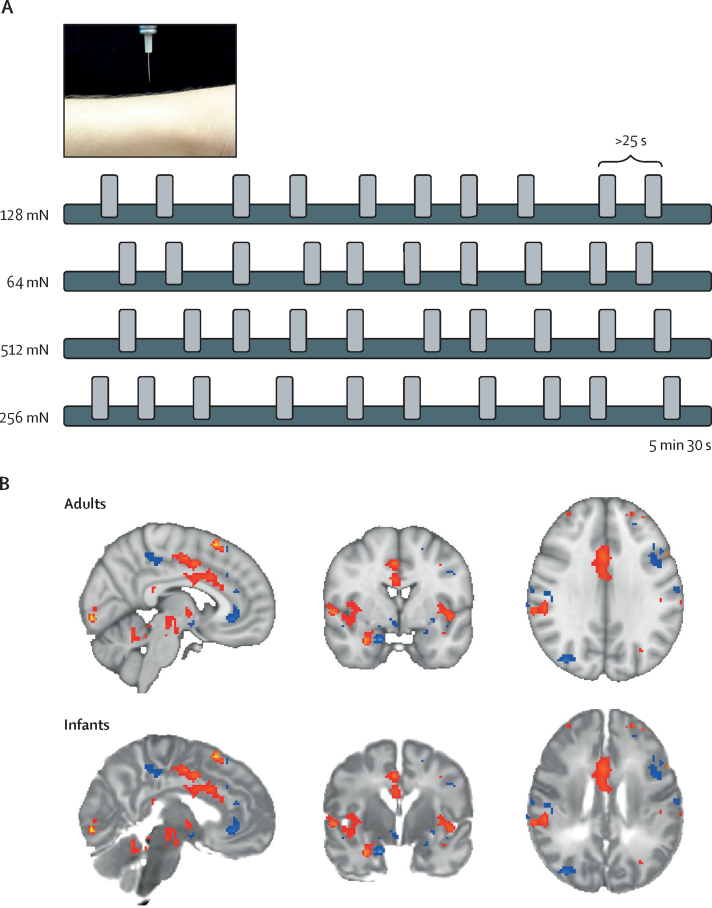
Experimental design and registration of the NPS in infants and adults (A) Stimuli were applied with a minimum interstimulus interval of 25 s across a range of intensities (64, 128, 256, or 512 mN) in a randomised order. Four stimulus intensities were used in adults (64, 128, 256, and 512 mN) and the two lowest stimulus intensities (64 and 128 mN) were used in infants. (B) The NPS was overlaid on the registered adult and infant templates, with both templates transformed into Montreal Neurological Institute template space (appendix p 2). Blue sections show anti-pain components of the NPS; red sections show pro-pain components of the NPS. NPS=neurologic pain signature.

All adults and infants in cohort A were scanned in a 3T Verio scanner (Siemens AG, Munich, Germany). Infants in cohort B were scanned in a 3T Prisma scanner (Siemens). Infants were transported to FMRIB 1–11 days after birth by clinical staff and parents were invited to accompany their child. Infants were wrapped, fed, and fitted with ear protection before scanning. The scan was stopped if infants became restless and re-initiated if deemed appropriate by clinical staff. Additional details of the fMRI procedures are provided in the appendix (p 1). During image acquisition, acute noxious experimental punctate stimuli of various intensities were applied to the left foot of each participant by a trained researcher. For each force, a train of ten stimuli was applied with a minimum interstimulus interval of 25 sec (figure 1A).

In adults, stimuli with forces of 64, 128, 256, and 512 mN were applied. In infants, only the 64 and 128 mN intensities were used to prevent behavioural distress and mitigate risk of tissue damage. The stimuli were applied when infants were naturally still. The nociceptive stimuli used in infants were rated as mildly painful by the adults. The intensity of the forces applied does not cause behavioural distress or evoke measurable clinical pain scores, in contrast to routine clinical heel lancing.^16^ However, these stimuli evoke reflex activity and electroencephalogram (EEG) activity in infants, which resemble activity elicited by more intense clinical procedures that break the skin, but of lower magnitudes,^17^ suggesting that the stimuli are mildly nociceptive in infants. No evidence suggests that the stimuli cause short-term or long-term harm or that repeated application results in sensitisation or habituation of reflex or brain activity.^16^

The order of scans was randomised across participants within each of the three cohorts and stimuli were time-locked to the fMRI recording using Neurobehavioural Systems software. After scanning, the stimuli were re-applied to the adults outside the scanner. In adults, pain intensity was reported verbally using the Numerical Rating Scale (NRS)^18^ after application of each stimulus. After all stimuli were applied, adult participants used the McGill Pain Questionnaire^19^ to describe their pain.

### fMRI brain signature analysis

All analyses were done in standard Montreal Neurological Institute template space, a commonly used template for the spatial normalisation of human brain images (figure 1B; appendix p 2). In the adult cohort, six brain signatures were applied to the fMRI response data, including the NPS^12^ and SIIPS1,^13^ and four control signatures: the vicarious pain signature,^11^ the picture-induced negative emotion signature (PINES),^9^ the social rejection signature,^14^ and the global signal signature. We analysed the correspondence of each signature to the parameter maps of each stimulus intensity using a modified pipeline from the Cognitive and Affective Neuroscience Laboratory (CANlab) toolbox (appendix p 2). Concordance between brain responses and signatures was defined by cosine similarity scores between the parameter maps and the predefined spatial signature images. Cosine similarity is equivalent to the Pearson correlation coefficient without mean-centring (range –1 to 1), and is the default similarity metric used in CANlab. The exclusion of mean-centring is useful for imaging signature analysis because it ensures the measure characterises similarity in both sign and magnitude.

The signatures were applied to the adult data to validate that signature responses were evoked following application of the mechanical noxious stimuli used in this study. To facilitate direct comparison of cosine similarities between adult and infant responses only signatures with significant concordance (p<0·05) in adults were applied to the infants. For each signature, a Spearman rank correlation test across stimulus intensities was used to assess intensity encoding of the responses.

The NPS extends across numerous brain regions and the strength of signature expression varies by spatial region.^12^, ^13^, ^20^ The NPS can be divided into a pro-pain subcomponent, in which the signature has a positive weight (ie, an increase in pain report is associated with increased brain activity) and a anti-pain subcomponent, in which the signature has a negative weight (ie, an increase in pain report is associated with decreased brain activity).^20^ Brain regions included in the pro-pain subcomponent correspond to known locations of nociceptive afferent pathways, and include the insular, somatosensory, and anterior cingulate cortices, and the anti-pain brain regions include the inferior parietal lobule and lateral occipital cortex.^20^ Both components track somatic pain, however, elements of the anti-pain subcomponent have been shown to be additionally responsive to the observation of pain in others.^11^ SIIPS1 has also been divided into pro-pain (including the insular cortex and central operculum), and anti-pain subcomponents (including areas of the somatomotor cortex, hippocampus, and cuneus).^13^ For SIIPS1, pro-pain regions have modest intensity encoding, whereas the majority of anti-pain regions do not.^13^ Therefore, we did secondary analyses to assess the concordance of pro-pain and anti-pain subcomponents of each signature, and subcomponents of these patterns. These subcomponents have been used in studies of the NPS^12^ and SIIPS1^13^ and were obtained using the CANlab toolbox. We adapted the CANLAB toolbox code to include regression of the NPS prior to SIIPS1, and the analysis of SIIPS1 subregions and single trial variability. Only brain regions that had nociceptive stimulus responses with non-negligible signature concordance (ie, mean cosine similarity >0·01) in the adult dataset were included in analyses.

### Role of the funding source

The funders of the study had no role in study design, data collection, data analysis, data interpretation, or writing of the report. The corresponding author had access to all the data in the study and was responsible for the decision to submit for publication.

## Results

Between May 22, 2013, and Jan 29, 2018, we recruited ten healthy participants to the adult cohort (five women and five men), 15 infants to infant cohort A (six girls and nine boys), and 22 infants to infant cohort B (11 girls and 11 boys). The mean age of the adult cohort was 28 years (range 23–36). The mean gestational age at the time of the study was 40·3 weeks (range 37–42) in infant cohort A, and 39·3 weeks (range 37–42) in infant cohort B, and mean postnatal age was 4 days (range 1–11) in infant cohort A, and 3 days (range 1–10) in infant cohort B.

**Figure 2:**
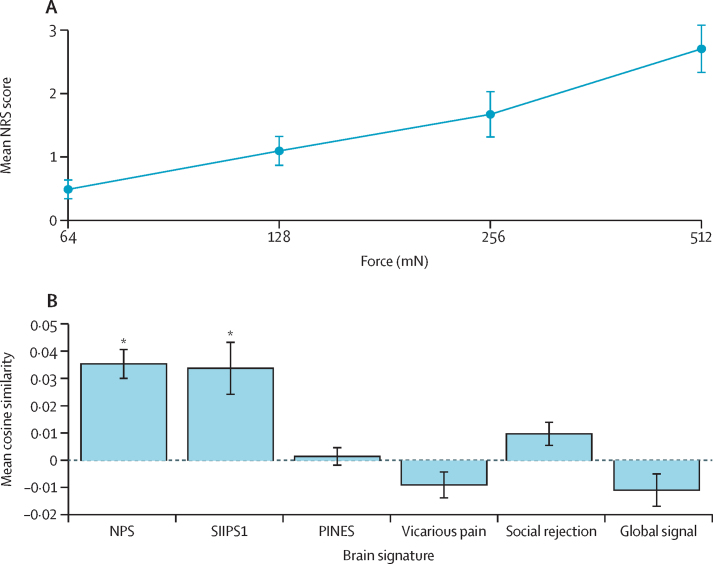
Intensity of the verbal pain report and quantification of the presence of six signatures in response to nociceptive input in the adult cohort (n=10) (A) Mean NRS scores with increasing stimulus intensity. (B) Mean cosine similarity for six signatures, including the NPS and SIIPS1, and four control signatures, in response to nociceptive input. Vertical lines show SE. NRS=Numerical Rating Scale. NPS=neurologic pain signature. SIIPS1=stimulus intensity independent pain signature-1. PINES=picture-induced negative emotion signature. *Cosine similarity significantly higher than zero.

Adults reported that the punctate stimuli evoked low-to-moderate pain, which they most frequently described as a pricking sensation (eight [80%] of ten participants) and a sharp sensation (six [60%] of ten participants), and verbal pain scores increased with stimulus intensity (*r*=0·89; 95% CI 0·80 to 0·95; p<0·0001; figure 2A),^6^ across a range corresponding to mild pain. All adults reported the 128 mN stimuli as mildly painful (NRS range 0·1 to 2·9). The NPS and SIIPS1 signatures were highly expressed in adults (the mean responses of ten [100%] of ten participants were positively associated with the NPS [cosine similarity >0]; the responses of nine [90%] of ten participants were positively associated with the SIIPS1 [cosine similarity >0]). However, significant expression of the four control signatures (vicarious pain, PINES, social rejection, and global signal) was not observed, confirming that brain activity evoked by the noxious punctate stimulus most closely resembled the neurologic and stimulus-independent pain signatures (NPS and SIIPS1; figure 2B). The NPS responses tracked the mean reported pain intensity in adults (*r*=0·61; 95% CI 0·37 to 0·77), similarly to previously reported results (*r*=0·74).^12^ The SIIPS1 signature was not significantly associated with verbal pain report (*r*=0·14; 95% CI –0·17 to 0·44; p=0·37).

**Figure 3:**
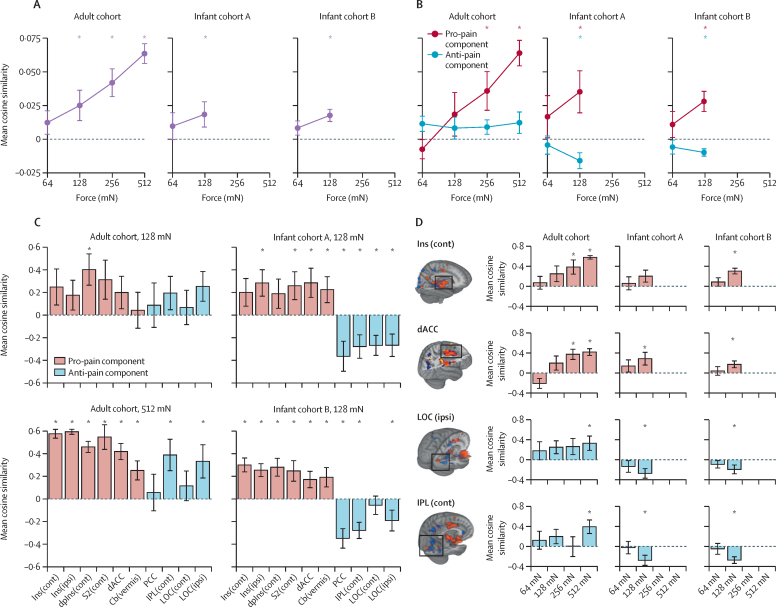
NPS responses (A) Mean NPS responses in the adult and infant cohorts with increasing stimulus intensity. All three cohorts had positive cosine similarities with evidence of intensity encoding. Vertical lines show SE. (B) Mean NPS responses in pro-pain and anti-pain components. (C) Mean NPS responses across different anatomical subregions following application of the 128 mN force (adult and infant cohorts) and 512 mN force (adult cohort only). Red bars show pro-pain components; blue bars show anti-pain components. (D) Intensity encoding of the NPS in selected subregions. Red bars show pro-pain components; blue bars show anti-pain components. NPS=neurologic pain signature. Ins(cont)=insula contralateral. Ins(ipsi)=insula ipsilateral. dpIns(cont)=dorsal posterior insula contralateral. S2(cont)=secondary somatosensory cortex contralateral. dACC=dorsal anterior cingulate cortex. cb(vermis)=cerebellum. PCC=posterior cingulate cortex. IPL(cont)=inferior parietal lobule contralateral. LOC(cont)=lateral occipital cortex contralateral. LOC(ipsi)=lateral occipital cortex ipsilateral. *Cosine similarity significantly higher than zero.

The NPS was consistently expressed in adults (mean cosine similarity of evoked brain activity greater than baseline brain activity, range 0·01–0·06), and showed significant intensity encoding between 64 and 512 mN (tested with a generalised linear model, p<0·0001; figure 3A), consistent with the verbal pain reports (figure 2A). Similarly, in infant cohorts A and B, application of the 128 mN stimuli evoked a change in brain activity concordant with the NPS (p=0·048 for infant cohort A; p=0·001 for infant cohort B; figure 3A). At 64 and 128 mN, mean cosine similarity values in infants were similar to adult scores (mean cosine similarity score range 0·012–0·024 for the adult cohort; 0·009–0·018 for infant cohort A; 0·008–0·017 for infant cohort B). In infants, the responses to 128 mN stimulation were greater than 64 mN stimulation in both cohorts; however, the differences were not significantly different in either cohort (figure 3A).

For the pro-pain NPS component, both adults and infants showed intensity-dependent activation (figure 3B), with greater concordance with the signature observed among infants than adults in response to the same intensity stimulation. In adults, subregion analysis showed positive mean responses in individual NPS pro-pain subregions, which were also consistently observed in infant cohorts A and B (figure 3C). Activity significantly increased with stimulus intensity within these brain regions in the adult cohort (p=0·030); a significant increase was not observed in infant cohort A (p=0·125) or infant cohort B (p=0·088). However, a significant increase was observed when data were combined across infant cohorts (p=0·034; figure 3D).

For the anti-pain subcomponent, the evoked activity in adults was concordant with the NPS, but was comparatively weaker than the pro-pain subcomponent (figure 3B). However, several individual anti-pain brain regions, including the inferior parietal lobule and the lateral occipital cortex, showed significant expression of the NPS in response to noxious stimulation (figure 3C, D). By contrast, infant cohorts A and B had negligible cosine similarity at 64 mN intensity, and significant negative cosine similarity at 128 mN (figure 3B). Regional analysis of the anti-pain brain regions that were significantly concordant with the NPS in adults found an absence of concordance in infants (figure 3C, D). An inverted response relative to the NPS template was observed in infants (figure 3C), corresponding to an increase in activation of these brain regions in response to noxious stimulation.

**Figure 4:**
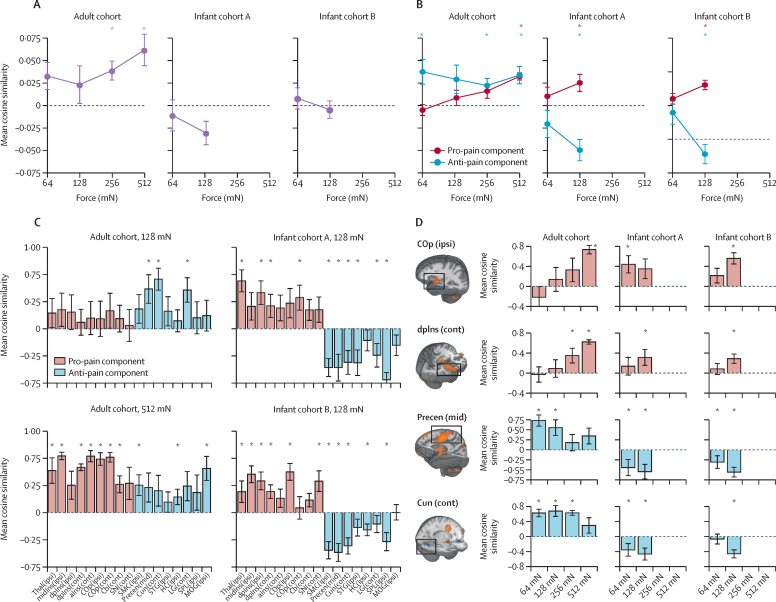
SIIPS1 responses (A) Mean SIIPS1 responses in the adult and infant cohorts with increasing stimulus intensity. (B) Mean SIIPS1 responses classified by pro-pain and anti-pain components. (C) Mean SIIPS1 responses across different anatomical subregions following application of the 128 mN force (adult and infant cohorts) and 512 mN force (adult cohort only). Red bars show pro-pain components; blue bars show anti-pain components. (D) Intensity encoding of the SIIPS1 in selected subregions. Red bars show pro-pain components; blue bars show anti-pain components. SIIPS1=stimulus intensity independent pain signature-1. Thal(ipsi)=thalamus ipsilateral. midIns(ipsi)=middle insula ipsilateral. dpIns(ipsi)=dorsal posterior insula ipsilateral. dpIns(cont)=dorsal posterior insula contralateral. aIns(cont)=anterior insula contralateral. COp(ipsi)=central operculum ipsilateral. COp(cont)=central operculum contralateral. Cb(cont)=cerebellum contralateral. SN(cont)=substantia nigra contralateral. SMC(ipsi)=somatomotor cortex ipsilateral. Precen(mid)=middle precentral gyrus. Cun(cont)=cuneus contralateral. STG(ipsi)=superior temporal gyrus ipsilateral. HC(ipsi)=hippocampus ipsilateral. LG(cont)=lingual gyrus contralateral. SPL(ipsi)=superior parietal lobule ipsilateral. MOG(ipsi)=middle occipital gyrus ipsilateral. *Cosine similarity significantly higher than zero.

SIIPS was reliably expressed in adults relative to baseline (mean cosine similarity score range 0·023–0·062) and was not significantly associated with intensity encoding (figure 4A). By contrast, noxious-evoked brain activity in infants was not concordant with the SIIPS1 signature, and in infant cohort A, negative concordance with the signature was observed when infants were stimulated with the 128 mN force (figure 4A). In the pro-pain subdivision of the signature, both adults and infants had a similar pattern of SIIPS1-related activation (figure 4B) and decomposition of the SIIPS1 pro-pain component indicated that brain regions reflecting the signature in adults showed similar responses in infants (figure 4C). However, negative concordance with the SIIPS1 was observed in both infant cohorts with 128 mN stimulation, which was opposite to that observed in adults (figure 4C). This divergence of adult and infant responses to the nociceptive input was observed across all subregions (figure 4C, D).

## Discussion

Multivariate fMRI signatures were designed to link features of brain activity to subjective experiences,^8^, ^12^ and a key potential application of these signatures is their use in patient populations with limited or no communication. In this study, we translated whole-brain fMRI signatures that predict aspects of pain perception in adults to the newborn infant brain, to advance understanding of how infants experience pain in early life. Data from our adult cohort confirmed that brain activity evoked by brief mechanical nociceptive stimulation was concordant with two validated brain signatures of pain, the NPS and SIIPS1. However, evoked brain activity was not concordant with control signatures of vicarious pain, negative affect, or global brain signals, providing evidence of the specificity of these signatures. In two independent infant cohorts, the NPS, a signature that tracks reported pain intensity across a variety of nociceptive stimuli in adults,^11^, ^12^ was significantly expressed in infant brain fMRI activity. However, the SIIPS1, which quantifies additional cerebral contributions to reported pain experience beyond nociceptive input and intensity encoding,^13^ was not observed in infants. This was likely driven primarily by a lack of concordance in the anti-pain brain regions. Furthermore, pro-pain NPS brain regions, primarily targeted by ascending nociceptive afferents, showed similar activation patterns in adults and infants, whereas anti-pain regions that are indirectly targeted by nociceptive afferents, showed divergent responses between adults and infants. These results suggest that the neural activity patterns that are indicative of intensity-modulated pain in adults, validated by correlation with verbal report, are present in infants.

The use of human language is an important method by which subtle sensory and affective states can be communicated. However, in non-verbal patient populations such as infants, inferences about sensory and emotional experiences are guided by behavioural and physiological observations. This approach is relied on clinically for the quantification and treatment of pain, but these measures are subjectively recorded and have poor specificity. Differentiation of pain from non-painful distress is challenging in infants, particularly when relying on behavioural responses.^21^ The association between observed behaviours and conscious pain experience is unknown. Neuroimaging provides more direct insight into the neural processes involved in infant experience and has the potential to improve inferences that can be made regarding pain and analgesia in non-verbal populations. In infants, noxious-evoked behaviour and brain activity have been studied and correlated.^22^, ^23^, ^24^ However, identification of a quantitative association between behaviour, or brain activity, and perception has remained elusive. In contrast to previous infant fMRI studies focusing on regional analyses of individual brain regions,^6^ multivariate fMRI signatures incorporating combinations of regional activity facilitate more accurate and specific prediction of pain report.^8^, ^13^

Our results provide quantitative evidence that noxious stimulation in infants evokes changes in brain activity that are attributed to the sensory discriminative aspects of pain experience in adults. Although infant brain responses to pain might reflect processes such as attention or stress, the NPS was developed to be specific to pain, involving structures directly targeted by nociceptive afferents, thus reducing the possibility that the infant responses reflect other processes. The NPS responses of adults showed significant intensity encoding. Although we did not observe a statistically significant difference in NPS cosine similarities for the two stimulus intensities in the infant cohorts, we did observe that higher intensity stimulation consistently evoked activity with higher cosine similarity for the NPS and pro-pain NPS subregions than did lower intensity stimulation. Considering that we observed a significant difference between the intensities for pro-pain NPS regions when infant cohorts were pooled, it is likely that this study was underpowered to detect a statistically significant difference to support intensity encoding within the individual cohorts. The similar expression of the NPS in both adults and infants, coupled with the differences in SIIPS expression across these cohorts, is consistent with results of a previous EEG study comparing adult and infant pain responses.^22^ In the study,^22^ infants displayed noxious-evoked potentials and γ oscillations, which are markers of primary nociceptive processing and subjective pain in adults.^25^

By contrast, nociceptive stimulation in infants did not evoke patterns of cerebral activity characterised by SIIPS1, which relate to complex psychological constructs in adults, including expectation, motivation, contextualisation, and perceived control of pain.^13^ During early development, infants become more engaged with their external environment, but it is not known at what developmental stage they are capable of interpretation, judgement, and modulation of their subjective experiences. Poor expression of the SIIPS1 signature might relate to the early stages of functional development of key brain regions such as the ventromedial prefrontal cortex and nucleus accumbens, and the limited, but developing ability of full-term newborn infants to engage these systems in processes such as descending modulation^5^, ^26^ and self-regulation.^27^ The absence of concordance of infant brain activity with SIIPS1 was primarily driven by divergent responses in anti-pain regions, which occurs when regions that are normally deactivated with increasing pain intensity are instead activated, for example in patients with fibromyalgia.^20^ Divergence of responses within the anti-pain subcomponent of the signature, including regions such as the cuneus, hippocampus, and areas of the precentral gyrus, was also observed in the NPS, providing the most marked axis of differentiation between infants and adults. The immaturity of the haemodynamic response in infants could produce altered blood oxygen level-dependent (BOLD) responses in these areas. However, although differences in BOLD mechanisms are observed in infant rats,^28^ we used a haemodynamic response function specifically derived from somatosensory responses in newborn human infants, which is only subtly different from the one used for adults.^29^ Alternatively, the divergence in anti-pain responses might be associated with true differences in noxious-evoked neural activity. For example, association and attentional networks are known to be immature in infants.^30^ The positive evoked BOLD response within anti-pain brain regions could be due to exuberant connections of the infant brain,^30^ coupled with a potentially underdeveloped GABAergic inhibitory system.^31^ Differences between infant and adult EEG responses to nociceptive stimuli have been reported, including delays in event-related potentials, and a late event-related potential component in infants corresponding to an increase in δ band energy.^22^ However, these explanations are speculative since the exact mechanisms underpinning differences in the evoked haemodynamic activity in the infant and adult brain remain unclear.

Although caution is advised when translating and interpreting adult signatures to the immature brain,^32^ considerable evidence exists to support the validity of this approach. Core structural features of the adult brain are developed in term-aged infants^33^ and functional responses,^34^ connectivity patterns, and resting state activity^35^, ^36^ recorded in infants have similar features to adult brain activity. Furthermore, marked neural activity overlap between adult and infant sensory-evoked and noxious-evoked brain activity patterns have been reported across several brain imaging modalities.^6^, ^22^ The lower effective spatial resolution in infant fMRI data results in poorer resolution of smaller brain structures in both the NPS and SIIPS1. Although these structures are important for pain perception, both signatures also have extensive contributions from larger brain structures that are well resolved in infants. For both the NPS and SIIPS1, previous studies have shown that signature subcomponents maintain their predictive capabilities,^11^, ^13^ suggesting that the signatures should maintain their association with pain perception in infants. This study has some limitations. Although the specificity of the signatures to pain has been validated in adults, establishing specificity in infants will require further investigation and the inclusion of additional control conditions. Furthermore, although these results were corroborated in two independent infant datasets, the relatively small sample sizes and limited range of low-intensity force that was applied to the infants meant NPS intensity encoding was not confirmed. The study was done in full-term neonates within a few days of birth, and therefore the results cannot be generalised to prematurely-born neonates or older infants.

Despite the strengths and weaknesses of fMRI, it is important to appreciate that a single modality cannot capture the full complexity of processes underlying a conscious experience. Therefore, we advocate a multimodal measurement approach to provide the best proxy of infant pain.^37^ Infant behavioural pain scores have been extensively studied, and brain-derived EEG signatures have been associated with infant nociception.^22^, ^23^ EEG-based measures have been validated for use as the primary outcome measure in clinical trials aiming to assess the efficacy of analgesic interventions,^17^, ^38^ and validation was achieved by considering the specificity of the EEG response to nociceptive input and characterising responses to established pain modulators. However, in the absence of verbal report, EEG-based measures cannot be directly associated with the infant's pain experience. By contrast, fMRI-based adult pain signatures provide the opportunity to combine distinct measures of activity across many elements of the pain system. Although it might be more challenging to resolve these contributions with EEG, it has superior high temporal resolution, low cost and ease of use in a clinical setting than fMRI. fMRI and EEG measures of pain-related brain activity are complementary and reflect distinct neural processes with different sensitivities to confounds and artifacts. Correlating the EEG measures to the fMRI signatures will enhance confidence in these approaches and help ensure that the best brain-derived surrogate pain measures are developed for use in non-verbal populations. Functional MRI is not a viable technique for measuring infant pain in clinical practice. Translation of these validated multivariate pain signatures to other modalities such as EEG will be important to maximise the clinical benefits of this work.

The translation of validated adult brain signatures to the immature brain has considerable potential. This study provides proof of concept for an approach to build more specific infant brain-derived biomarkers of pain. Considering the clear structural and functional similarities in the organisation of the full-term newborn infant and adult brain, projecting brain signatures onto the infant brain, which have been refined on the basis of adult verbal pain descriptors, improves the inferences that can be made about infant pain. A potential application would be to compare the signature representation in infants born at full term and infants born preterm who have reached term to assess how early life experiences associated with premature birth affect pain experience. There is also value in applying the signatures to older children (age ≥6 years) capable of verbally reporting their pain, to investigate the developmental trajectory of the signatures. These signatures could be further optimised for infants by testing and validating them in adults in studies using experimental designs that replicate the reduced contextual understanding experienced by infants.^39^

This study expands the use of brain signatures to infer and deconstruct the experience of pain in a non-verbal patient population. Identifying the best proxy to quantify pain experience in infants is crucial for the management of pain in neonatal care and the mitigation of potential long-term and short-term effects of pain in early life. This work provides a framework to better understand the early development of human sensory and emotional experience and provides a potential novel approach to assess the effect of analgesic interventions in infancy.

## Data sharing

Authors will share analytic code and raw, de-identified individual participant brain imaging data on request with researchers who provide a methodologically sound proposal and can conduct analyses that achieve the aims of the proposal. We are preparing a data sharing platform and associated study protocols and code, which should be completed approximately 3 months post publication. Data sharing requests can be directed to rebeccah.slater@paediatrics.ox.ac.uk. To gain access data requestors will need to sign a data access agreement.
