# Supplementary material for: Inferring pain experience in infants using quantitative whole-brain functional MRI signatures: a cross-sectional, observational study
Source: Lancet Digit Health. 2020 Aug 24;2(9):e458–67. doi: 10.1016/S2589-7500(20)30168-0 (PMC7480713; doi:10.1016/S2589-7500(20)30168-0)
Supplement: Supplementary appendix [file mmc1.pdf]

THE LANCET
Digital Health

Supplementary appendix

This appendix formed part of the original submission and has been peer reviewed. We post it as supplied by the authors.

Supplement to: Duff EP, Moultrie F, van der Vaart M, et al. Inferring pain experience in infants using quantitative whole-brain functional MRI signatures: a cross-sectional, observational study. *Lancet Digital Health* 2020; **2:** e458–67.

Supplementary Material (Methods)

MRI acquisition

For adults, BOLD images were acquired using a T2*-weighted EPI acquisition (sequence parameters: TR/TE = 3280/40ms; flip angle = 90°; FOV = 192×192 mm; imaging matrix 64×64; resolution 3×3×3 mm; slices = 50, collected in descending order; average total volumes = 142). For Infant Cohort A, the same fMRI sequence was used with a reduction in slices (33 slices) and an infant-optimised TR/TE of 2500/40ms [1]. For Infant Cohort B: BOLD images were acquired using a T2* BOLD-weighted, GRE acquisition with EPI readout, 70° flip angle, TE= 50 ms[1], TR= 1,300 ms, mean TA= 6 mins (approx.), multiband 4[2,3], 90 x 90 in-plane matrix size, 56 slices, 2 mm isotropic voxels. All studies used a 32-channel head coil.

MRI Pre-processing

All infant fMRI data were processed using the dHCP functional pipeline[4,5] Motion and distortion correction of infant BOLD fMRI data in the dHCP fMRI preprocessing pipeline is necessarily more extensive than in typical adult pipelines, due to the substantial image contamination that occurs due to infant head motion. Using the dHCP pipeline, our infant data were head motion corrected for both volume misalignments due to between-volume motion and slice misalignments due to within-volume motion, known as slice-to-volume effects. Additionally, the dynamic distortion correction accounted for the magnetic field changes that occur due to certain head rotations, known as susceptibility-by-motion effects. These advanced motion and distortion corrections were simultaneously performed using the FSL tool EDDY[6,7], as implemented in the dHCP fMRI pipeline. ICA-based denoising was subsequently performed on the corrected data in a similar manner to the adult pipeline outlined in the main text[8,9] For the infant data, the subject head motion parameter timeseries were estimated using the DVARS metric[10], which is based on the rate of change of signal from volume-to-volume and closely reflects subject head motion as well as other artefacts. A 3mm low-pass spatial filter and 100s period high-pass temporal filter were applied.

The adult data were processed using FSL 6.0[11]. BOLD data were motion and distortion corrected to remove volume misalignment and susceptibility-induced distortions, high pass temporally filtered with a 100s cut-off period to remove slow temporal drifts, and low-pass spatially filtering with a 5mm FWHM kernel to remove high frequency spatial noise. The data were decomposed using spatial independent component analysis (ICA) to identify detrimental structured signal components due to both biological and scanner-related sources. Standard fMRI ICA-based clean-up was employed, which simultaneously regressed from the BOLD data both ICA noise components and subject head motion parameter timeseries (estimated during motion correction).

The pain responses were modelled using a general linear model (GLM) in FEAT[11] in which the expected stimulus-response BOLD timeseries for the sequence of stimuli is regressed onto the data independently at each voxel. The expected timeseries was derived for adults by convolving the experimental design (a timeseries

defining the specific application of the stimuli) with the canonical adult double gamma BOLD haemodynamic response function, and for infants by convolving the experimental design with the term-infant-specific optimal basis functions generated by Arichi and colleagues[12]. Using a summary statistics approach[1311], regression parameter maps were generated for each adult and infant at each stimulus intensity and transformed into standard Montreal Neurological Institute (MNI) template space for further analysis, via a series of transformations (Fig. 1B; see below) to account for individual variability in morphology.

Regression parameter estimate maps for individual subject and intensity levels were transformed into the standard adult MNI space via a series of transformations. This step was necessary so that the adult-derived signatures could be projected onto the infant brain. Alignment between infant functional (BOLD) and structural (T2) space was performed using boundary-based-registration (BBR), which aligned the boundary between cerebral grey matter and white matter. Alignment between infant structural space and the standard age-matched T2 template[14] was performed via non-linear registration. Week-to-week nonlinear transforms registered the infant data from the age-matched template to the 44-week template. To align the infant 44-week template with the adult MNI template, the NIHPD2 lifespan atlases (ages birth to 21 years) were used as intermediate non-linear transform targets[15,16]. All infant non-linear transforms were performed using ANTs's SyN (Advanced Normalization Tools Symmetric image Normalization method), using multimodal registration with both T1 and T2 structural template images to best account for the changing structural image contrast over development[17]. All transforms between infant functional space and adult MNI space were combined and applied in a single step to minimise image degradation due to resampling and interpolation. For adults, FNIRT nonlinear registration was used to transform data to MNI space[13].

Signature analysis

The CANlab toolbox provides a pipeline for processing, quality control and visualisation of parameter maps for the signature-based analysis of our group fMRI data. This included the extraction, assessment, regression and of white matter and cerebro-spinal fluid components, the calculation of signature similarity measures, estimation of contrasts and SVM classifier maps, and NPS subregion analysis. Parameter maps from each of the three cohorts were organised for processing with the CANlab toolbox. The signature response was estimated for each participant in each test condition by calculating the cosine similarity of vectorised activation images and template signature maps, masked for brain tissue only. As a measure of similarity, cosine similarity will be dependent on the spatial properties of the signatures and effective spatial resolution, which will differ between adults and infants and across signatures. Therefore, we do not directly compare similarity measures across groups.

The SIIPS1 signature was trained using data from which the contribution from NPS had been removed via regression[19]. We performed the same procedure here, removing the NPS spatial signature from the GLM

response parameter maps. We adapted CANLAB code to include regression of the NPS prior to SIIPS1, and analysis of SIIPS1 subregions and single trial variability https://github.com/canlab.

Response statistics and intensity encoding were assessed using a two-level GLM summary statistics approach, modelling participants as random effects[13].

Supplementary References

1 Goksan S, Hartley C, Hurley SA, *et al.* Optimal echo time for functional MRI of the infant brain identified in response to noxious stimulation. *Magnetic Resonance in Medicine* 2017; **78**. DOI:10.1002/mrm.26455.

2 Moeller S, Yacoub E, Olman CA, *et al.* Multiband multislice GE-EPI at 7 tesla, with 16-fold acceleration using partial parallel imaging with application to high spatial and temporal whole-brain fMRI. *Magnetic Resonance in Medicine* 2010; **63**: 1144–1153.

3 Xu J, Moeller S, Auerbach EJ, *et al.* Evaluation of slice accelerations using multiband echo planar imaging at 3T. *NeuroImage* 2013; **83**: 991–1001.

4 Baxter L, Fitzgibbon S, Moultrie F, *et al.* Optimising neonatal fMRI data analysis: Design and validation of an extended dHCP preprocessing pipeline to characterise noxious-evoked brain activity in infants. *Neuroimage* 2019; **186**: 286–300.

5 Fitzgibbon SP, Harrison SJ, Jenkinson M, *et al.* The developing Human Connectome Project (dHCP) automated resting-state functional processing framework for newborn infants. *bioRxiv* 2019; 766030.

6 Andersson JLR, Graham MS, Drobnjak I, Zhang H, Filippini N, Bastiani M. Towards a comprehensive framework for movement and distortion correction of diffusion MR images: Within volume movement. *NeuroImage* 2017; **152**: 450–466.

7 Andersson JLR, Sotiropoulos SN. An integrated approach to correction for off-resonance effects and subject movement in diffusion MR imaging. *NeuroImage* 2016; **125**: 1063–1078.

8 Griffanti L, Salimi-Khorshidi G, Beckmann CF, *et al.* ICA-based artefact removal and accelerated fMRI acquisition for improved resting state network imaging. *NeuroImage* 2014; **95**: 232–247.

9 Salimi-Khorshidi G, Douaud G, Beckmann CF, Glasser MF, Griffanti L, Smith SM. Automatic denoising of functional MRI data: combining independent component analysis and hierarchical fusion of classifiers. *NeuroImage* 2014; **90**: 449–68.

10 Power JD, Barnes KA, Snyder AZ, Schlaggar BL, Petersen SE. Spurious but systematic correlations in functional connectivity MRI networks arise from subject motion. *NeuroImage* 2012; **59**: 2142–2154.

11 Jenkinson M, Beckmann CF, Behrens TEJ, Woolrich MW, Smith SM. FSL. *NeuroImage* 2012; **62**: 782–90.

12 Arichi T, Fagiolo G, Varela M, *et al.* Development of BOLD signal hemodynamic responses in the human brain. *Neuroimage* 2012; **63**: 663–73.

13 Mumford JA, Nichols T. Simple group fMRI modeling and inference. *Neuroimage* 2009; **47**: 1469–75.

14 Schuh A, Makropoulos A, Robinson EC, *et al.* Unbiased construction of a temporally consistent morphological atlas of neonatal brain development. *bioRxiv* 2018; : 251512.

15 Evans AC. The NIH MRI study of normal brain development. *NeuroImage* 2006; **30**: 184–202.

16 Fonov V, Evans A, Mckinstry R, Almli CR, Collins L. Unbiased nonlinear average age-appropriate brain templates from birth to adulthood. *Neuroimage* 2009; **47**. DOI:10.1016/S1053-8119(09)70884-5.

17 Avants BB, Epstein CL, Grossman M, Gee JC. Symmetric diffeomorphic image registration with cross-correlation: evaluating automated labeling of elderly and neurodegenerative brain. *Medical Image Analysis* 2008; **12**: 26–41.

18 Andersson J, Jenkinson M, Smith SM. Non-linear registration, aka spatial normalisation. *FMRIB technical report TR07JA2* 2007; published online June.

19 Woo C-W, Schmidt L, Krishnan A, *et al.* Quantifying cerebral contributions to pain beyond nociception. *Nat Commun* 2017; **8**: 14211.
